# Use a ball-ended anterior cruciate ligament reamer to protect patella tendon during minimal access tibial nailing

**DOI:** 10.1308/003588412X13373405385214s

**Published:** 2012-07

**Authors:** J Granville-Chapman, DS Elliott

**Affiliations:** Ashford and St Peter’s Hospitals NHS Foundation Trust,UK

**Figure 1:**
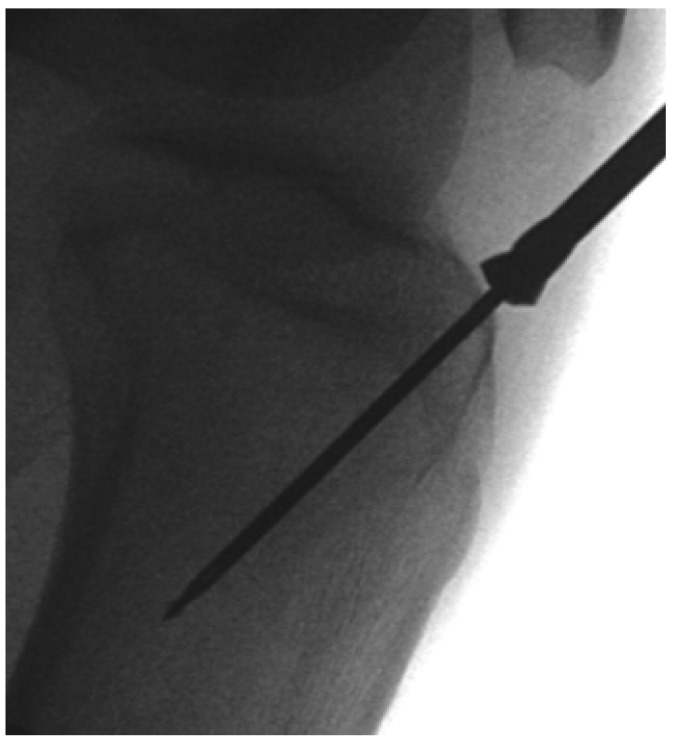
Ball-ended 10mm anterior cruciate ligament reamer

Minimal access tibial nailing is popular. After entry point identification and guidewire placement, a cannulated entry reamer is used to ‘open’ the medullary canal. Rather than using the manufacturer’s guidewire and bulky 12mm reamer, we recommend using the anterior cruciate ligament tibial guidewire and the 10mm ball-ended reamer (Fig 1).

This reamer’s short cutting length minimises damage during reaming of proximal tibial metaphysis as the reamer can be pushed gently through the incised tendon before it is activated. The 10mm hole in metaphyseal bone allows easy passage of subsequent canal reamers and the tibial nail.

